# Supplementary material for: Astemizole Sensitizes Adrenocortical Carcinoma Cells to Doxorubicin by Inhibiting Patched Drug Efflux Activity
Source: Biomedicines. 2020 Jul 29;8(8):251. doi: 10.3390/biomedicines8080251 (PMC7460240; doi:10.3390/biomedicines8080251)
Supplement: Supplementary file 1 [file biomedicines-08-00251-s001.pdf]

biomedicines

Astemizole Sensitizes Adrenocortical Carcinoma Cells to Doxorubicin by Inhibiting Patched Drug Efflux Activity

Anida Hasanovic, Méliné Simsir, Frank S. Choveau, Enzo Lalli and Isabelle Mus-Veteau

Supplementary Figures

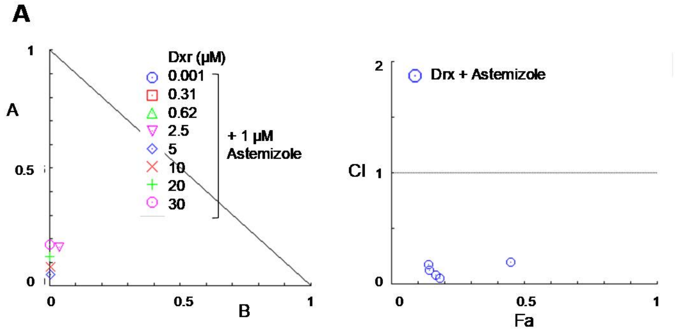

Fa	Dose Dxr	Dose Ast	DRI Dxr	DRI Ast
0.95	0.13746	0.18403	137.463	0.18403
0.95	0.13746	0.18403	0.44343	0.18403
0.95	0.13746	0.18403	0.22171	0.18403
0.451	15.1875	27.3126	6.07499	27.3126
0.184	105.316	213.859	21.0633	213.859
0.168	124.253	254.943	12.4253	254.943
0.145	161.369	336.571	8.06844	336.571
0.14	171.569	359.225	5.71898	359.225

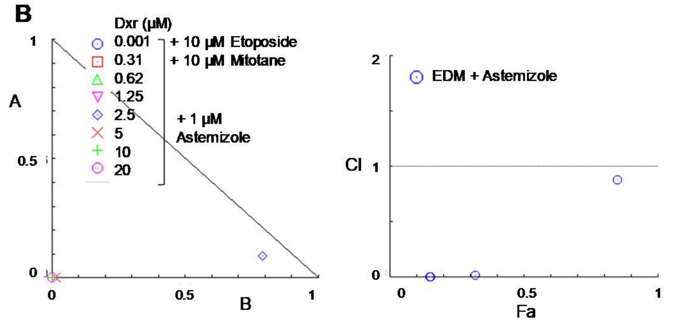

Fa	Dose EDM	Dose Ast	DRI EDM	DRI Ast
0.99	0.02116	0.01330	21.1636	0.01330
0.99	0.02116	0.01330	0.06827	0.01330
0.99	0.02116	0.01330	0.03413	0.01330
0.99	0.02116	0.01330	0.01693	0.01330
0.85	27.3814	1.26262	10.9526	1.26262
0.32	13946.9	66.3015	2789.38	66.3015
0.152	156501.	308.177	15650.1	308.177
0.15	162738.	315.926	8136.92	315.926

Figure S1. Drug combination synergism. (**A**) Between doxorubicin and astemizole. (**B**). Between EDM and astemizole. The normalized isobologram (left), the combination index plot (middle) and the corresponding dose-reduction index (DRI) table (right) are presented. Fa, fraction affected. The diagonal line represents a cooperativity index of 1 indicating additive interaction of the combined treatment. The drug combinations below the curve indicate synergism.

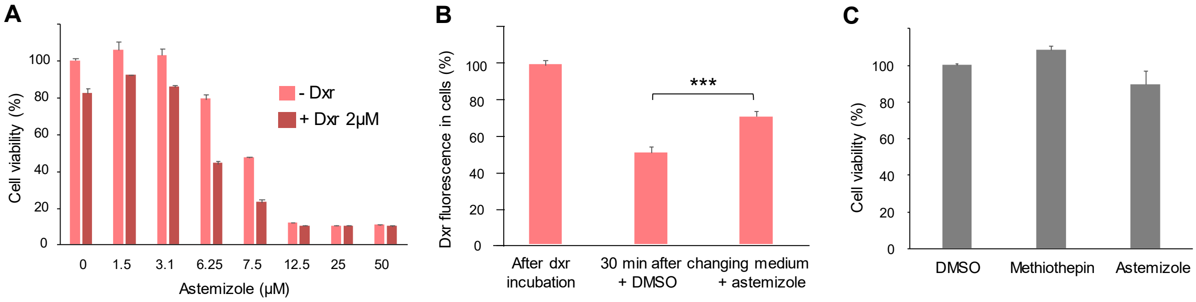

Figure S2. Astemizole effect on melanoma cells and keratinocytes. (**A**). Astemizole increases the cytotoxic effect of doxorubicin in melanoma cells. Cell viability was measured after 48 hours treatment of MeWo cells with serial dilutions of astemizole in the presence or the absence of doxorubicin (dxr) 2 µM. The graphs reported are representative of more than 3 independent experiments. (**B**). Astemizole inhibits the efflux of dxr in melanoma cells. MeWo cells were seeded on coverslips and incubated with dxr. After 2 hours, 3 coverslips were fixed for dxr loading control. The other coverslips were incubated with DMSO or astemizole 30 µM for 30 min and fixed. Images were acquired and dxr fluorescence was quantified using Image J software for 100 cells per condition. (**C**) Astemizole slightly affects keratinocyte viability. Cell viability was measured after 48 hours treatment of HaCaT keratinocytes with DMSO, 10 µM methiothepin or 10 µM astemizole. Histograms represent the mean ± SEM values of 3 independent experiments. Significance, calculated using ANOVA multiple comparison test and Bonferroni correction, was attained at $p < 0.05$ (*) (***: $p < 0.0005$).
